# A Brief Resume of the Grosser Animal Nature and Its Application in Medicine

**Published:** 1901-05

**Authors:** G. N. Jack

**Affiliations:** Depew, N. Y.


### A BRIEF RESUME OF THE GROSSER ANIMAL NATURE AND ITS APPLICATION IN MEDICINE.[1]

By G. N. JACK, M. D., Depew, N. Y.

(*Concluded from April number.*)

ANOTHER, and one of the most fruitful sources for the parasitic growth of quacks, and in fact the weakest point in our profession today, lies in the changeable, unstable and unsettled condition of our etiology and treatment.

The etiology and treatment of disease is the part of our science that most directly concerns the layman, and leaves us open for attack.

If the etiology and treatment of disease could be brought down to bare undisguised nature, like our embryology, anatomy, physiology, pathology and chemistry are today, the physician would stand high in the community, and proudly feel that he possessed a superior knowledge which gave him peculiar rights that no one could infringe upon.

We have but very little positive knowledge as to the etiology of disease. Many theories have been advanced, but theories prove nothing. Theories that are advanced today and contradicted tomorrow produce uncertainty, and uncertainty breeds contempt.

The layman, through our newspapers, ever eagerly post themselves on the numerous contradictory, conflicting and disappointing theories that are continually being advanced, thus encouraging them to believe that the medical fraternity are possessed of no real, actual, well-established and practical knowledge. To allow such a spirit to exist among the laymen is dangerous, for it often creates in them a feeling of distrust, or still worse, gives them the erroneous opinion that the regular practitioner is no better than the willfully, lying, deceiving, advertising quack.

Has our present popular germ theory of disease come to stay, or is it like theories of the past, doomed sooner or later to gradually sink into oblivion? I will not attempt to answer that question in this paper, but I will simply give you a few thoughts for consideration.

The germ theory of disease has no attraction for a close observer of nature and nature's phenomena, for every phenomenon that this theory attempts to explain, can be proven by chemical facts. No one can dispute, however, but that the germ theory has been a great boom to the animal kingdom, for with it came cleanliness, and cleanliness will wash away precipitate and destroy a chemical poison as

1. Read at the annual meeting of the Medical Society of the County of Erie, January 8th, and at the Central Medical Association, Lancaster, N. Y., February 4, 1901.

well as an imaginary germ. If we consider this disease producing factor as a chemic rather than a germic substance, it so gracefully and securely glides into the machinery of nature as to defy all authority or further speculation to the contrary. All life, growth, and in fact nature itself, is a bi-product and a continuation of chemical reactions and equasions.

It is conceded by all that healthy, normal animal or vegetable life is a result of and directly dependent upon a fixed, definite, positive and accurate set of chemical reactions that admit of slight variations as to age, surroundings and habits. It follows, then, as a natural and indisputable law that all diseases, with their various degrees and manifestations, must be due to an abnormal biochemy.

Nature offers numerous illustrations of this law, one of which is the changes life undergoes under different temperatures and atmospheres. For instance, in a torrid temperature, under a bright sun and a clear atmosphere, we meet with chemical changes producing sunstroke. In the same temperature, with a damp, cloudy, humid atmosphere, combined with the influence of rapidly decomposing animal and vegetable matter, we meet with a different chemical change, resulting in a disintegration of the blood, characterised as all chemcal actions are, by, first, an abstraction of heat or chill, and, second, by a precipitation, increased heat, or fever. These chemical actions we see most strikingly demonstrated in malarial fever.

Now, if we pass to the temperate region, with a chilly, damp, cloudy, humid atmosphere, we meet with a different set of biochemical changes which acts more on the chemistry of digestion than on that of the blood, producing that condition known clinically as "la grippe."

By passing on to the frigid regions, we again meet with still another abnormal biochemical process, manifesting itself clinically, first, by a tingling, prickling sensation; second, by a chill; third, by numbness; fourth, by drowsiness, and fifth by death. If this process be stopped at the chill it may be followed by a healthy reaction and do no harm, but it not infrequently combined with the irritating effect of the inhaled cold air upon the respiratory apparatus, results according to the susceptibility of the individual into either a coryza and a bronchitis or a pneumonia. If an illy protected portion of the body, as a toe or a finger, be frozen sufficiently to produce death of that member, it will, if amputated soon, do no further harm, but if this frozen member be left and allowed to decompose *in situ*, by so doing a cadaveric alkaloid will be formed that may by absorption, produce a progressive inflammation and necrosis, to continue until the entire vitality of the body is exhausted.

The germ theory of disease could be bunglingly crowded into these arguments, but the most careless student of nature can hardly fail to see how simple uncomplicated and substantial, our knowledge of disease would become by the exclusion of the germ idea.

If an overdose of the chemical poison known as strychnine be injected into the circulation, it produces a characteristic set of muscular spasms and death, and so if the chemical poison generated from a rusty nail in the horse stable be introduced into the circulation, it produces nearly the same muscular contractions and death in tetanus. This thought can be applied to all of our supposed germic inoculations, the toxins and antitoxins of today.

In order to prove that mildew, mold, fermentations, putrefactions, or slow decomposition are of germic origin, it will be necessary to prove also that fire, or rapid decomposition, together with all our different chemical reactions are of the same origin, for they are all governed by the same law, belong to the same class, and ultimately produce the same results. The only difference is that one group has a slow, the other a rapid process.

Life in life, which is the fundamental principle of the germ theory, is not in its actual, visible forms, very, if at all, injurious to health. In fact nature has made provision for life to exist in life; thus we find the fetus normally growing and developing for nine months in utero, and during five months of this time it is an active, kicking, squirming urchin, without injuring, but usually actually benefiting the health of the mother. Nature does not stop here even, but provides for another twelve months parasitic life for the infant upon the breast of the mother.

A most convincing, if not positive proof, of the incorrectness of the germ theory, lies in the fact that all the numerous parasites, whether antecious, heterecious, accidental, andophytic or epiphytic will feed and thrive on, or in living animals without in many instances doing any harm to the health of their host, and when they do harm it is always of a mechanical and never of a toxic nature.

Thus we find the filaria sanguinis hominis doing harm by its actual presence, obstructing the lymph channels. The coenurus cerebralis produces staggers by actually burrowing into the brain and spinal marrow. The ankylostomum duodenale, produces anemia by robbing its host of food which it absorbs for its own sustenance, and also by reflexly causing dyspepsia.

Now, in consideration of the fact that animals may and often do harbor numerous parasites with comparatively little, if any detrimental results, it is certainly, to say the least, farfetched and not in accord with nature, to argue that an invisible imaginary germ will so greedily destroy life.

By excluding the germ theory our etiology and prophylaxis would be greatly simplified, and the unknown origin of many diseases determined. We then could speak of vaccination as introducing a chemical substance to produce a chemical change. Immunity in general could be reasoned out and demonstrated on a scientific basis, thus furnishing tangible material for the construction of an artificial immunity in all diseases, characterised by a permanent chemical change that protects against subsequent attacks as scarlet fever, measles and syphilis.

Our reason, then, for quarantineing would be to keep the unafflicted from coming in contact with a chemical poison that produces characteristic and deleterious results.

With all due regard for the earnestness, intelligence, well-meaning and high-standing of the originators and perpetuators of the germ theory of disease, yet, with nature as a judge, one cannot help but acknowledge a striking similarity between the aboriginal medicine man, whose function it was to drive away evil spirits from the afflicted, and the physicians of today who feel that it is their duty to protect their patients from invisible, hungry germs, that are ever hovering near, and impatiently awaiting an opportunity to devour them.

Our present treatment of disease, including both surgery and therapeutics, is also in a very unsettled and unsatisfactory condition, yet it has a redeeming feature in that there is a strong tendency to a just appreciation and recognition of nature and her laws. Physicians and surgeons themselves are usually conscious of nature's superiority over drugs, or the knife, in all cases where she can be utilised, yet they will, not infrequently, purposely deceive or sanction the self-deception of their patients, by prescribing and operating according to the delusive opinions and demands of the layman. Deception is dangerous, and those who attempt to profit by it will more than once feel the sting of the lash they unwittingly try to wield.

The medical fraternity often give drugs that they have no faith in, or use the knife for mental effect, and at the same time order a hygiene that they have confidence will and usually does cure their patient, thus giving the layman the idea that the sole function of the physician is either to disfigure or poison. That this spirit is prevalent among the laymen is alarmingly manifested by the increasing patronage of the numerous "no knife," "no drug cure" quacks of the day. The laymen are not, as the medical fraternity is apt to assume, all fools, but, on the contrary, they eagerly keep abreast of the times, and in every possible manner inform themselves on such vital questions as beauty or disfigurement, health or sickness, life or death.

With our excellent public educational advantages, and the excessively large number of physicians, many of whom are constantly and publicly disagreeing with each other, we are safe to assume that the laymen are reasonably accurate judges of the true and the false in medicine.

Essential progressive experimentation should ever be encouraged and boldly practised, but the surgeon who habitually dissects all those complaining of vague pains, to the extent of his ability and the patient's vitality, to conclude that his patient's organs are normal, but her mind hysterical; or he who unnecessarily suspends kidneys; removes appendices, ovaries or uteruses, is gradually committing a professional suicide. Surgeons should be exceedingly cautious in operating upon cases that could be permanently cured by an intelligent application of Nature's laws.

Park, from his extensive investigations of malignant growths will, in their case, soon settle the question between the knife and medicinal applications. Halstead, with his careful history of all cases, both before and after operating, will soon settle the question between the knife and nature. There is a great sufficiency of unquestionably beneficial surgery to occupy a reasonably large number of surgeons without manufacturing cases.

Last spring at the Johns Hopkins Hospital, I heard Osler advise, while holding a clinic over a case of moveable kidney, never to let a neurasthenic know that she has a movable kidney, for she will not rest until she reaches the surgeon, and many of these cases can be cured by a bandage with increased fat, and he jocundly added, "the lay papers take these subjects up, they are tired of appendicitis, the tail has wagged the dog long enough, they will now take up moveable kidney."

To come back to the medical side, we find numerous physicians who are continually endeavoring, and earnestly believing, that it is their duty to substitute drugs for nature, or, in other words, to do with drugs what nature and nature alone can do. Then, again, there are many physicians who think that they must give drugs to collect their fees, but utilise nature to produce the cure. This is a mistake, for it gives their patients an erroneous idea which, when discovered, drives them to the no-drug quack. There are enough drugs whose actions are positive and often proven, that when intelligently used as nature's assistant will save life, relieve suffering, and restore health, to never justify their deceptional use to cheat nature. There is no excuse for our carrying diamonds in our pockets and wearing stones of paste on our breasts. Most people will willingly pay for advice, but hesitatingly spend their money for drugs.

We find Osler boldly combating many diseases with no other weapon than a due regard and careful consideration for nature's laws. Thus he successfully treats two of our most dreaded diseases —consumption and typhoid fever—without, in many instances, the aid of a single drug. I have constructed a hygiene for the asthmatic that has proven to be very beneficial, in that, theretofore, hopelessly considered, no-use-of-doctoring-sort of a disease.

Advertising patent medicine liars, have taught the laymen to believe that the physician should possess a secret and a positive cure for each and every affliction. The physicians should ever endeavor to correct that dangerous opinion. Show them that as there is no secret to the tools or material that a carpenter uses in constructing houses, yet the most fool-hardy, would never dream of constructing one of our modern dwellings without the special knowledge, skill and training of a full-fledged carpenter. Let them know that, so it is with the physician, for while he possesses no secret remedies, nor practises any mysterious art, yet through his profound knowledge of histology, physiology, anatomy, pathology, chemistry and physical diagnosis, he is enabled to construct laws of life for each individual case, and to intelligently apply the common so-called household remedies, which together restore health, relieve suffering, and save life. Yet, none but those possessed of the knowledge of the physician, can ever hope to achieve the beneficial results with these agents that the physician himself can accomplish. Point out to them the fact that a certain amount of doctoring is natural and essential to the perfect development of all animal life, for the cat when sick will take its catnip; the horse will nibble the bitter cherry bark, the cow will eat the bitter burdock, and the like.

It is my custom, with the ordinary neurasthenic office patient, if after a thorough examination, I find that I can be substantiated in doing so, to state to her that I can cure her with or without the socalled drugs, but that I prefer to give a little medicine as a "starter" and to let nature do the rest. I then proceed to impress upon her the importance of the advice, I am about to impart, by informing her of the countless years of profound study and research that it has cost the medical fraternity, to be able to positively ascertain the things which are best suited to her case, and which, if scientifically applied, will cure her. I show her that the entire chemistry of the body must be changed, and that in order to perfect this change it will require approximately three months' diet, salt rubs, cold spinal douches, cold sponge baths, massage and exercise treatment. And in order that I may know what part of the treatment to occasionally change, and when best to do so, to bring it to a scientific and satisfactory issue, I advise her to call once in three days, during these three months, thus enabling me, in her presence, as the occasion demands, to analyze her urine, feces, blood, muscular tone and reflexes, which serves, first, to keep me scientifically in touch with the case; second, to indelibly impress upon my patient the importance of the analysis, and, third, it gives her confidence in the directions I give, which makes her faithful to their fulfillment.

Thus it is that as one engineer may run an engine into the shop as worthless, a more skilled engineer will often take the condemned engine, and without any material repairs, will by first shining the engine up to make it proud and gain its confidence, and, second, by knowing the proper quantity, quality and time for coaling; when to shake down; when to shovel out; when and what to oil; when and how to take on water; and how best to regulate the draft; thus admirably causing her to usefully run out the natural period of her existence.

Just so a careless and unscientific physician will often give up a case as hopeless, to be taken up by a careful and scientific physician who will, by gaining confidence, and scientifically knowing, when, what and how to feed; when and how to bathe; the kind of exercise or recreation needed; proper clothing; and a correction of all vicious habits; thus by using nature as a guide, nurse the case back to health, vigor and usefulness.

In conclusion, I would say, let us not deceive nor be deceived, but boldly take off our coats and undisguisedly meet nature face to face, thus showing our noble character, and causing, in the minds of the laymen, our superior knowledge to tower so high that the parasitic quack may never hope to shadow its brilliancy.

